# Effects of epidermal growth factor on the invasive activity and cytoskeleton of oral squamous cell carcinoma cell lines

**DOI:** 10.3892/ol.2014.1946

**Published:** 2014-03-07

**Authors:** YUICHI OHNISHI, MASAHIRO WATANABE, HIROKI YASUI, KENJI KAKUDO

**Affiliations:** Second Department of Oral and Maxillofacial Surgery, Osaka Dental University, Chuo-ku, Osaka 540-0008, Japan

**Keywords:** epidermal growth factor, matrix metalloproteinases, matrix metalloproteinase 1, squamous cell carcinoma, cytokeratin 19

## Abstract

Epidermal growth factor (EGF) is present at high concentrations in human saliva and modulates the growth and differentiation of various cancer cells. To elucidate the molecular mechanisms by which EGF affects oral cancer proliferation and invasion, the current study analyzed the Matrigel invasion activity of cultured oral cancer cell lines. Cell proliferation under the influence of EGF was subjected to Matrigel invasion assays, and cell proliferation in the absence of EGF was used as control. Northern blot analyses quantified the invasiveness and tumorigenicity. Chloramphenicol acetyltransferase assay determined the EGF stimulation of matrix metalloproteinase (MMP) 1 expression. EGF increased the number of cells penetrating the Matrigel membrane. Northern blot analysis revealed that MMP1 and cytokeratin 19 expression correlate with EGF. In addition, the morphology of HSC-3 and SAS cells changed following the addition of EGF to the culture medium. A transient transfection assay revealed that EGF increases the promoter activities of MMP1 in HSC-3 cells. These observations suggested that EGF increases the invasive activity of oral cancer cells, partly by increasing MMP1, and morphological changes may be induced by altering the composition of cytoskeletal proteins.

## Introduction

In 1962, Cohen identified epidermal growth factor (EGF) while studying nerve growth factor in submaxillary glands of male Swiss-Webster mice (1). EGF is known to be produced in human salivary glands. It modulates the proliferation and differentiation of various cancer cells, as well as normal epithelial cells. EGF has been shown to enhance bladder cancer cell growth (2–4). Furthermore, it is known that EGF stimulates matrix metalloproteinases (MMPs), which have been previously reported to be associated with metastases in various tumors (5–7). Advances in molecular biology have revealed that MMPs are particularly important in cancer progression (8), and the expression levels of MMPs correlate with cancer invasion and metastasis (9). We previously reported that EGF increases the promoter activities of the MMP9 gene in HSC-3 cells (10). These results suggested that EGF increases the invasive activity of oral cancer cells partly by increasing MMP9 (10).

Cytokeratin (CK) 19 is a particularly intriguing member of the CK intermediate filament superfamily (11). Unlike other type I acidic CKs, CK19 does not generally form heterodimers with any of the type II basic proteins (12). CK19 is expressed in the non-keratinizing stratified squamous epithelium exemplified by the oral cavity (13) and tends to reflect the state of differentiation of the tumor (14). For example, the expression of CK19 is more intense in poorly differentiated lesions than in other lesions. In a previous study, keratin 19 downregulation in oral squamous cell carcinoma cell lines increased their invasive potential (15).

The present study analyzed the effects of EGF on the invasive activity of a cultured oral cancer cell line and assessed the transcription of MMP1. In addition, the expression of CK19 with and without EGF was examined.

## Materials and methods

### Cell culture

The human oral squamous cell carcinoma (SCC) cell lines, HSC-3, SAS and Ca9-22, were obtained from the RIKEN BioResource Center (Ibaraki, Japan). HSC-3, SAS and Ca9-22 cells were cultured in Dulbecco’s modified Eagle’s medium (DMEM; Nissui, Tokyo, Japan) containing 10% fetal bovine serum (FBS) and 5 μg/ml ampicillin at 37°C in humidified 5% CO_2_ in air. Human recombinant EGF (Upstate Biotechnology, Lake Placid, New York, USA) was added to the medium at concentrations of 0, 10 and 100 ng/ml, and the medium was changed every day. Following EGF addition, cell lines were cultured for six days.

### Observation of cell morphology

HSC-3 or SAS cells (15×10^4^) were seeded in 1 ml DMEM containing 1% FBS and EGF (0, 10 or 100 ng/ml) in a 35-mm dish for six days, during which the medium was changed every day.

### Matrigel invasion assay

The assays were performed according to the manufacturer’s instructions. In conclusion, HSC-3 and SAS cells (2×10^4^/well) were seeded in a six-well Bio Coat Matrigel^®^ Invasion Chamber (Beckton-Dickinson, Bedford, MA, USA) in Eagle’s minimal essential medium containing 10% (v/v) heat-inactivated fetal calf serum, with 0, 10 and 100 ng/ml EGF. Following 48 h of incubation, the non-invading cells were removed from the upper surface of the membrane by scrubbing and the membrane was stained using a Diff-Quick stain kit (Sysmex, Kobe, Japan). Subsequently, invading cells on the lower surface were counted using a microscope (BX50, Olympus Corporation, Tokyo, Japan). Data are presented as the average of experiments performed in triplicate.

### RNA isolation and northern blot analysis

HSC-3 and SAS cells (30×10^4^) were seeded on a 100-mm gelatin-coated dish. Following 48 h of incubation, EGF was added to the medium at a concentration of 10 ng/ml and the medium was changed every other day. Total cellular RNA was prepared following six days of culture using the Isogen^®^ RNA isolation kit (Nippon Gene, Tokyo, Japan). For northern blot analysis, 15 μg of total RNA was electrophoresed on formaldehyde-agarose gel in 3-(N-morpholino)propanesulfonic acid buffer and transferred to nitrocellulose filters (Nitroplus-2000, Micron Separations Inc., Westborough, MA, USA) in 20X SSC (1.5 M NaCl and 0.15 M sodium citrate). The filters were prehybridized in 50% formamide, 4X SSC, 5X Denhardt’s solution [0.1% Ficoll (GE Healthcare Japan Corporation, Tokyo, Japan), 0.1% polyvinylpyrrolidone (Sigma-Aldrich Japan K.K., Tokyo, Japan) and 0.1% bovine serum albumin (Takara Bio Inc., Shiga, Japan)], 0.2% sodium dodecyl sulfate (SDS; Wako Pure Chemical Industries, Ltd., Osaka, Japan) and denatured sonicated salmon sperm DNA (120 μg/ml; BioDynamics Laboratory Inc., Tokyo, Japan), at 42°C for 2 h and hybridized with radiolabeled DNA probes (CK19, EGFR and GAPDH) under the same conditions for 24 h. Following hybridization, the filters were washed at 55°C with 0.1X SSC containing 0.1% SDS.

### DNA probes

A human CK19 cDNA clone in pUC18 (*Eco*RI cloning site; 1.5*-*kb insert) and a human EGF receptor (EGFR) cDNA clone in pBR322 (*Cla*I cloning site; 2.4-kb insert; pE7) were used as probes and a human glyceraldehyde 3-phosphate dehydrogenase cDNA clone in pUC18 (*Eco*RI cloning site; 1.2-kb insert) was used as a control probe.

### Western blot analysis

Lysates obtained from 2.0 μg/ml CRN197-treated HSC-3 cells incubated for 24 h were resuspended in 0.2 ml radioimmunoprecipitation assay buffer (Wako Pure Chemical Industries, Ltd.) (0.1% Nonidet-P40, 1 mM CaCl_2_, 1 mM MgCl_2_, 0.1% sodium azide, 1 mM phenylmethylsulfonyl fluoride, 0.03 mg/ml aprotinin and 1 mM NaVO_4_). Proteins were separated on 7 and 14% SDS polyacrylamide gels (Nacalai Tesque, Inc., Kyoto, Japan), transferred overnight at 20 V and incubated for 2 h at room temperature with mouse monoclonal antibody, followed by incubation with secondary horseradish peroxidase-conjugated antibodies (Anti-Mouse IgG, whole Ab from Sheep; GE Healthcare UK Ltd., Amersham, UK) at a dilution of 1:1,000. Primary mouse monoclonal antibodies included anti-MMP1 (Calciobiochem, Darmstadt, Germany). Immunoreactivity was detected using enhanced chemiluminescence. Densitometric analysis was performed with ChemiDoc using Quantity One software (Bio-Rad, Tokyo, Japan).

### Chloramphenicol acetyl transferase (CAT) assay

In summary, MMP1-CAT was constructed by ligating a 563-bp fragment (−518 to +45 of MMP1 genomic DNA) to the bacterial CAT assay performed in HSC-3 cells using a transient transfection system (Lipofectamine™; Invitrogen Life Technologies, Carlsbad, CA, USA). Cells (1.5×10^6^) were seeded in a 100-mm dish and 10 μg of DNA was co-transfected with 5 μg of pCH110 plasmid as an internal control using the calcium phosphate co-precipitation method. The culture medium was changed 16 h following transfection to 10% DMEM with or without 10 ng/ml EGF. A cell lysate was prepared 48 h following transfection and the reaction was performed. Data are presented as the average of experiments performed in triplicate.

### Statistical analysis

The Mann-Whitney U test was used to assess the statistical significance of differences between samples. P<0.05 was considered to indicate a statistically significant difference.

## Results

### Expression of CK19 and EGFR mRNA in HSC-3 and SAS

**Figure 1:**
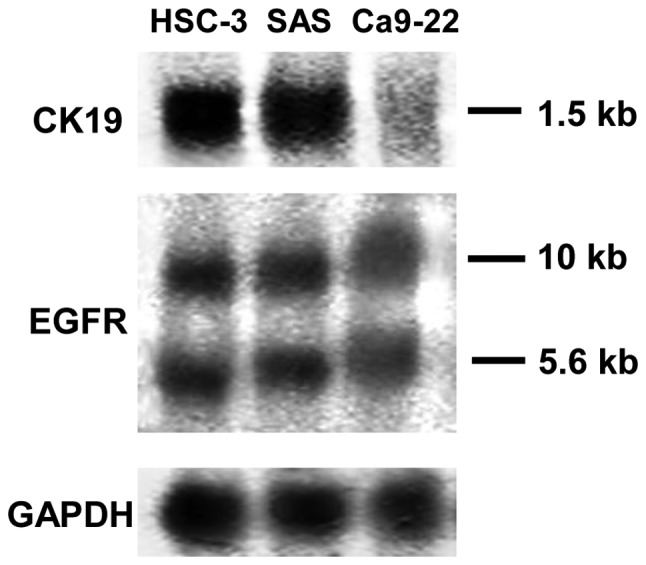
Northern blot analysis of CK19 and EGFR mRNA in oral squamous cell carcinoma cell lines. In total, 10- and 5.6-kb EGFR mRNA and 1.5-kb CK19 mRNA were detected in HSC-3, SAS and Ca9-22 cells. CK19, cytokeratin 19; EGFR, epidermal growth factor receptor.

EGF stimulates the proliferation of target cells through interaction with its surface receptor. Previous studies have shown that EGFR mRNA is expressed in a large number of oral SCC cell lines. In total, 10- and 5.6-kb EGFR mRNA was detected in the present study from HSC-3, SAS and Ca9-22 cells. This result confirmed the observations of previous studies for HSC-3 cells, but this is the first report of EGFR mRNA detection in SAS cell lines. Similar levels of expression of EGFR mRNA were detected in HSC-3 and SAS cells (Fig. 1).

### Morphological change in HSC-3 and SAS in medium containing EGF

**Figure 2:**
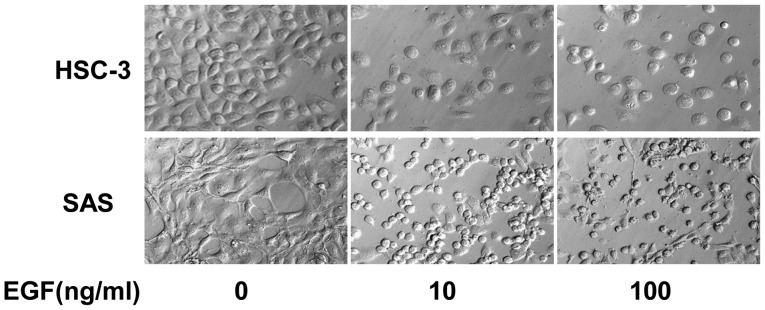
Morphological changes in HSC-3 and SAS cells. Cells were cultured in medium containing 0, 10 and 100 ng/ml EGF for six days. Images were captured using light microscopy at a magnification of ×40. EGF, epidermal growth factor.

EGF produces various changes in cells. Therefore, morphological change in HSC-3 and SAS was observed under EGF stimulation. HSC-3 and SAS exhibited typical proliferation in the absence of EGF. By contrast, HSC-3 and SAS exhibited morphological change in the presence of EGF. It was shown that their form changed to circular; in addition, cells showed detachment, with the loss of cell adhesion. Fig. 2 shows the morphology of HSC-3 and SAS on day six. As bladder cell carcinoma cell lines piled up into discrete colonies due to EGF, differences were identified among the oral SCC cell lines. This suggested the possibility of variable cell morphological changes among the cell lines under EGF stimulation. These observations suggested that EGF affects the degree of cell-cell adhesion of tumor cells.

### Invasiveness and tumorigenicity

**Figure 3:**
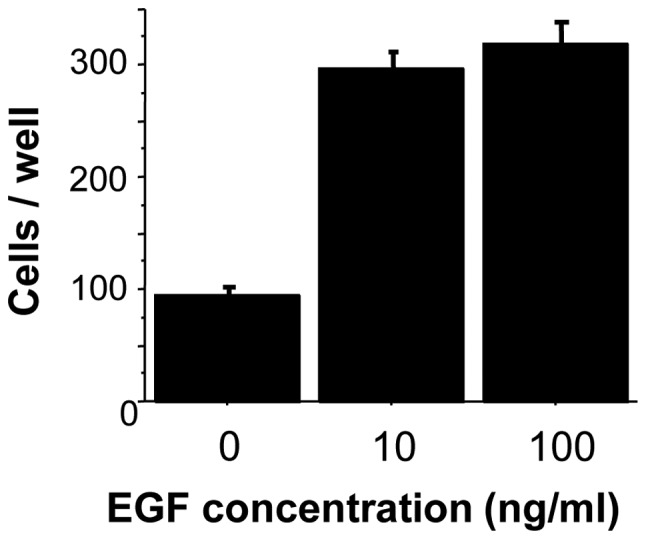
EGF-enhanced invasive activity of oral cancer cells. The number of HSC-3 cells penetrating the Matrigel membrane was approximately three-fold higher in EGF-stimulated (10 ng/ml) cells than in unstimulated HSC-3 cells. The numbers of HSC-3 cells stimulated with 10 and 100 ng/ml EGF were almost the same. Data are presented as the mean ± standard errors of triplicate eperiments. EGF, epidermal growth factor.

To determine whether EGF increases the invasive activity of oral cancer, the invasiveness of EGF-stimulated HSC-3 cells was investigated using Matrigel invasion chambers that included extracellular matrix components. The number of HSC-3 cells penetrating the Matrigel membrane was approximately three-fold higher in EGF-stimulated (10 mg/ml) cells than in unstimulated cells. No significant difference was identified in the penetration of HSC-3 cells with the various concentrations of EGF used (10 and 100 mg/ml) (Fig. 3).

### Northern blot analysis

**Figure 4:**
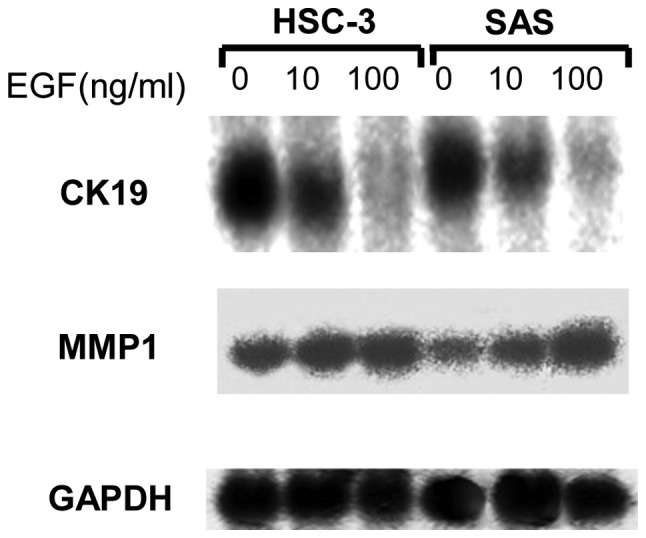
Expression of MMP1 and CK19 mRNA in northern blot analysis. EGF increased the expression of MMP1. By contrast, the expression of CK19 mRNA was evidently reduced by EGF, even at the lowest concentration of 10 ng/ml. GAPDH mRNA was used as an internal marker of applied RNA amounts. EGF, epidermal growth factor; MMP1, matrix metalloproteinase 1; CK19, cytokeratin 19; GAPDH, glyceraldehyde 3-phosphate dehydrogenase.

Northern blot analysis was used to analyze the influence of EGF on the expression of MMP1. EGF increased the expression of MMP1. Furthermore, the expression of CK19 was analyzed to investigate the correlation between cell conformation and CK expression. The expression of CK19 mRNA was evidently reduced by EGF, even at the lowest concentration of 10 ng/ml (Fig. 4).

### MMP1 expression in western blot analysis

**Figure 5:**
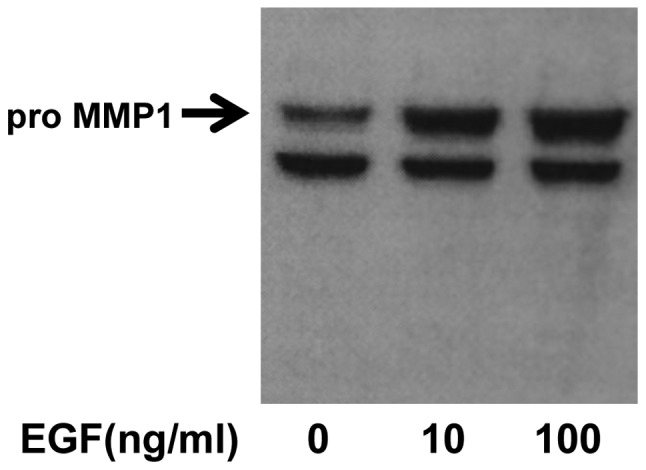
MMP1 expression in western blot analysis. To confirm an increase in MMP1 protein levels, western blot analysis was performed using an anti-MMP1 monoclonal antibody. EGF increased the expression of MMP1 protein in an EGF concentration-dependent manner. EGF, epidermal growth factor; MMP1, matrix metalloproteinase 1.

To confirm an increase in MMP1 protein levels, western blot analysis was performed using an anti-MMP1 monoclonal antibody. EGF increased the expression of MMP1 in an EGF concentration-dependent manner (Fig. 5).

### EGF stimulates MMP1 expression

**Figure 6:**
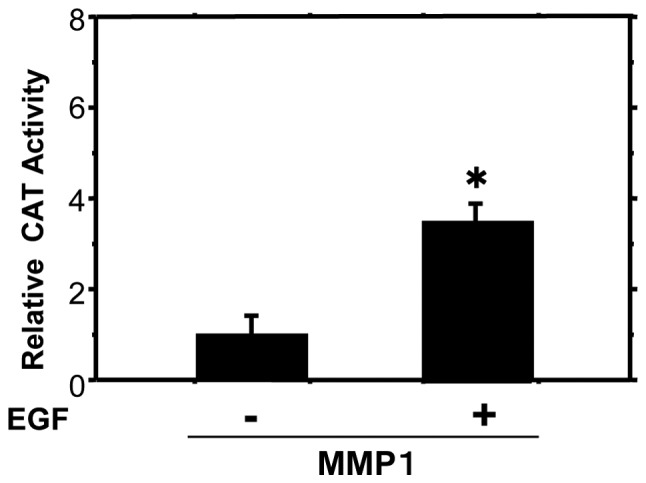
Effect of EGF on MMP1 promoter activity. Reporter plasmids containing the MMP1 promoter (MMP1-CAT) were co-transfected with CH110 as an internal control into HSC-3 cells. Cells were placed in 10% Dulbecco’s modified Eagle’s medium with 10 ng/ml EGF 16 h following transfection. CAT activity, measured 48 h following transfection, was corrected for variations in the internal control and is presented as the relative activity. MMP1-CAT activity was increased 3.7-fold by EGF stimulation compared with that in controls. Data are presented as the mean ± standard errors of triplicate experiments. EGF, epidermal growth factor; MMP1, matrix metalloproteinase 1; CAT, chloramphenicol acetyltransferase.

As EGF increased the invasiveness of the HSC-3 oral cancer cell line, it was assessed whether it is also likely to modulate the expression of MMP1. CAT reporter genes driven by MMP1 promoters were transiently transfected into HSC-3 cells. MMP1-CAT activities were increased 3.7-fold following EGF stimulation compared with the controls (Fig. 6).

## Discussion

MMPs are considered to be important in cancer invasion and metastasis (8,16,17). MMPs degrade various types of collagen, namely, stromal type 1 collagen and type 4 collagen of the basement membrane, and proteoglycans. The expression of matrix-degrading proteinases is elevated in a variety of invasive carcinomas (18,19). However, the activating mechanisms of these invasion-associated genes remain poorly understood. Certain previous studies have shown that EGF stimulates the expression of MMP1 and MMP9 in several cancers (16–18). In the present study, EGF stimulated HSC-3 and SAS cells to invade Matrigel membranes. Additionally, EGF stimulated HSC-3 and SAS cells to produce MMP1 mRNA. Notably, it has been previously reported that SCC exhibits overexpression of EGFRs (18,19). These previous studies suggest that EGF and EGFRs are likely regulatory factors for the production of MMP1. In addition, the present study found by CAT assay that EGF stimulates the MMP1 promoter. These observations suggested that the stimulation of MMP1 expression by EGF is likely to be one of the mechanisms that increases the invasive activity of HSC-3 and SAS cells.

CK8, CK18 and CK19 are CKs that are part of the cell skeleton and arrange the cell conformation similar to actins. CKs are also involved in maintaining desmosomal junctions through connections with certain cadherins, including desmogleins and desmocollins. Thus, CKs are essential for cell-cell adhesion similar to E-cadherin. In addition, CK8 and CK18 have been previously reported to be expressed at high levels in various cancers (20). Certain previous studies have reported the reduced expression of CK19 in poorly differentiated skin and breast cancer (21). The observations of the current study showed that EGF reduced the expression of CK19 mRNA in oral cancer cell lines. Reduction of CK19 may be an additional pathway through which EGF increases the invasive activity of oral cancer cell lines.

The standard care for oral cancer is a combination of surgery, radiation and chemotherapy. Commonly, carboplatin, cisplatin and paclitaxel are used for chemotherapy. However, in specific patients, these drugs have no effect due to the development of drug resistance. In oral cancer, overexpression of EGFR has been associated with chemoresistance and poor prognosis. Targeting EGF, as an abundant EGFR ligand, may be favorable for the treatment of chemoresistant oral cancer. It has also been noted that EGF inactivates CK19. Therefore, EGF may be a better target for the therapy of oral cancer than EGFR.

In conclusion, the current study identified two pathways of EGF effects on oral carcinoma cell lines. Firstly, that the expression of MMP1 results in degradation of the extracellular matrix. Secondly, that changes in cytoskeletal proteins, such as CK19, may allow changes in morphology that promote movement into vessels and lymphatics. The results of the present study suggested that EGF may promote the invasive activity of oral cancer cells by activating these two pathways.
